# CO_2_ emission and socioeconomic inventories of Guangdong-Hong Kong-Macao Greater Bay Area and surrounding cities 2000-2022

**DOI:** 10.1038/s41597-025-06076-4

**Published:** 2025-11-14

**Authors:** Ya Zhou, Pingda Lu, Aiqun Guan, Yuli Shan, Dabo Guan, Zhifeng Yang

**Affiliations:** 1 Guangdong Basic Research Center of Excellence for Ecological Security and Green Development, Key Laboratory for City Cluster Environmental Safety and Green Development of the Ministry of Education, School of Ecology, Environment and Resources, Guangdong University of Technology, Guangzhou, 510006 China; 2 School of Geography, Earth and Environmental Sciences, University of Birmingham, Birmingham, B15 2TT UK; 3 Department of Earth System Sciences, Tsinghua University, Beijing, 100080 China

**Keywords:** Environmental impact, Climate-change mitigation

## Abstract

The Guangdong-Hong Kong-Macao Greater Bay Area (GBA) is a leading economic region and a pilot demonstration region of carbon peaking in China. The city-level time-series CO_2_ emission inventories of the GBA region are crucial for the formulation of policies on climate change mitigation pathways. However, the region lacked a consistent and comparable time-series city-level CO_2_ emissions inventory dataset. In this study, we provided CO_2_ emission and socioeconomic inventories of the GBA cities and their surrounding twelve Guangdong cities from 2000 to 2022. The CO_2_ emission inventories were compiled by 47 economic sectors, 17 types of fossil fuels, and four industrial processes. The dataset provides temporal emissions estimates that support the design of regions’ mitigation strategies, and help China achieve its goal of peaking carbon emissions before 2030.

## Background & Summary

**Fig. 1:**
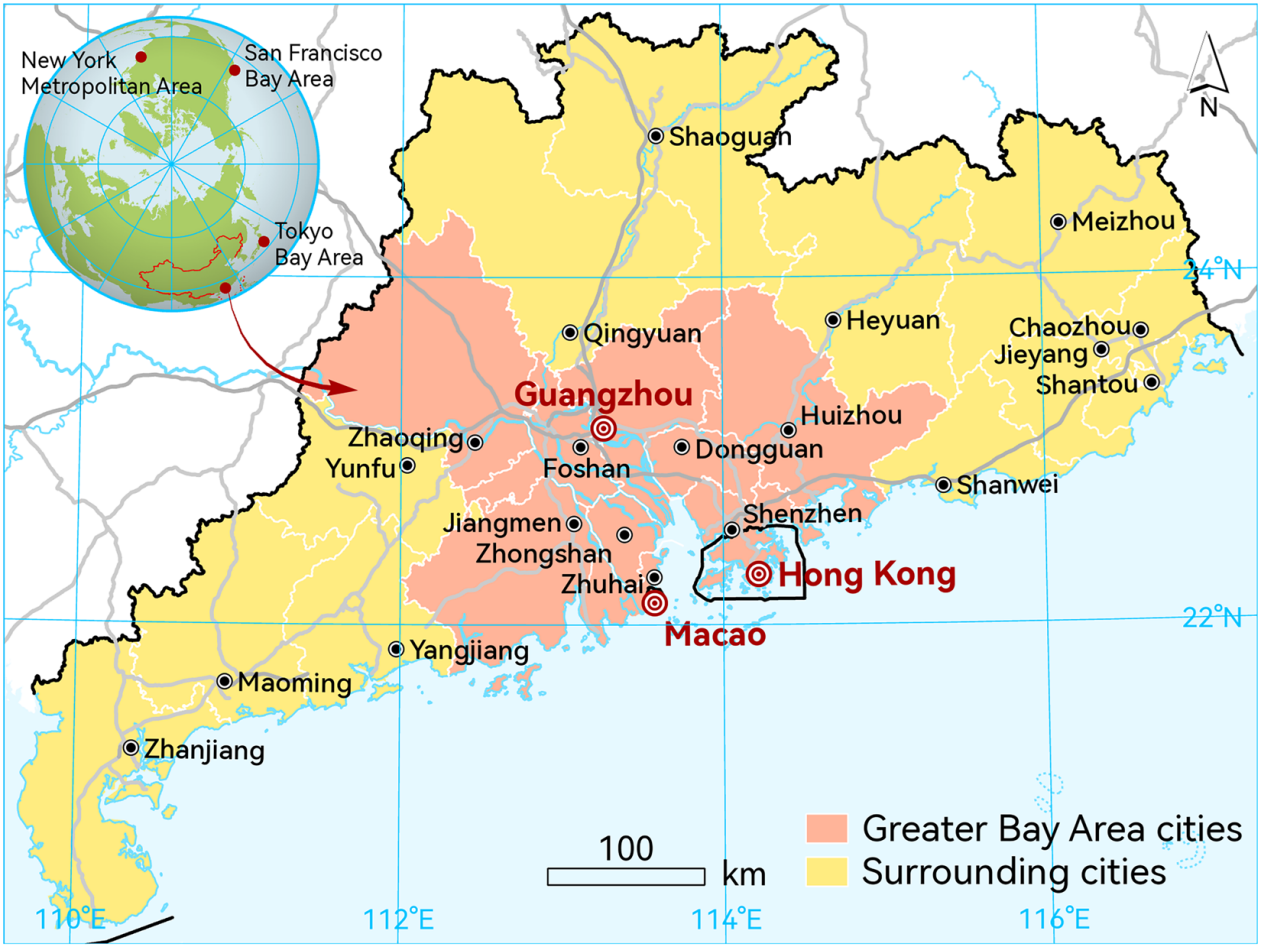
Geolocation of the Guangdong-Hong Kong-Macao Greater Bay Area and surrounding cities.

Cities are responsible for over 70% of global carbon dioxide (CO_2_) emissions from energy consumption^1^, highlighting their critical importance in addressing climate change and emissions reduction. The Guangdong-Hong Kong-Macao Greater Bay Area (GBA) in southern China, featuring rapid urbanization and world-class city clusters, is at the forefront of promoting comprehensive green transition in economic and social development^2,3^. The GBA consists of nine Guangdong Province cities, Hong Kong, and Macao (Fig. 1). This region contributed 11% of the national Gross Domestic Product (GDP) in 2024 with only 0.58% of the territory and 6% of the population^4^. The other twelve Guangdong cities surrounding the GBA had close ties with the GBA cities in terms of industry and infrastructure^5^. In 2022, the GBA’s GDP growth rate of 9.3% ranked first among the four globally prominent bay areas, followed by the New York Metropolitan Area (7.2%), the Tokyo Bay Area (3.5%), and the San Francisco Bay Area (3.3%)^6–8^. But the energy consumption per unit of GDP in GBA was higher than the three bay areas’ average due to high economic growth^9^. With the continuous growth of population and economy, the energy consumption and resource pressure in GBA were expected to increase^10^. Adapting to global climate challenges, the Chinese government set ambitious goals to peak carbon emissions by 2030 and reach carbon neutrality by 2060, and the GBA was identified as one of the pilot demonstration regions for peaking carbon emissions and achieving carbon neutrality^3^. The GBA has taken measures to reduce CO_2_ emissions, including replacing fossil fuels with clean energy and optimizing the industrial structure^11^.

Consistent, comparable, transparent, and time-series emission inventories are crucial for city-level decision-makers to assess the effectiveness of emission mitigation efforts and develop targeted climate action plans through identifying key emission sources. Existing studies have estimated CO_2_ emissions of GBA but mainly focused on specific socioeconomic sectors, such as residential sector^12^ and power generation^13^. Some studies focused on individual core cities^14,15^ or specific years^16–18^, limiting the understanding of time-series variations in carbon emissions. The comparability of some cities’ emission inventories is limited due to inconsistencies in accounting system boundaries and emission factor selections^16,17,19–21^. Some studies estimated city-level CO_2_ emissions with proxy data (e.g., GDP, night-time light imagery, building morphology)^12,15,19,21^, which may overlook sectoral information that helps identify key emission contributors (Table 1).

**Table 1:** Previous studies on carbon dioxide emission accounting in the Greater Bay Area.

Ref.	Case City	Emission scope	Emission calculation	Sector	Time-span
^12^	GBA and surrounding Guangdong cities	CO_2_ emissions from in-boundary energy consumption, and imported electricity generation	Cities with energy balance table: sectoral approach, with emission factors collected from Shan *et al*.^28^; Cities without energy balance table: downscaled from estimations with nighttime light data	Residence	2010, 2020
^13^	GBA cities	CO_2_ emissions from energy consumption by electricity generation and purchased electricity	Emission factor method with emission factors collected from Ministry of Ecology and Environment^51^	Electricity generation	2020
^14^	Shenzhen	CO_2_ emissions from energy consumption	Emission factor method, with emission factors collected from CDMC^52^, IPCC^26^, and South China Power grid	Public transport	2005–2015
^15^	Hong Kong	CO_2_ emissions from energy consumption, electricity, and heating generation	Sectoral approach with emission factors collected from IPCC^26^, downscaled to 100m-grid with proxy data (transport network and building morphology)	Residence, business, industry, and transport	2016
^16^	305 Chinese cities (including GBA and surrounding Guangdong cities)	CO_2_, CH_4_, N_2_O, fluorinated GHGs from in-boundary energy consumption and industrial processes, and imported electricity (only CO_2_ covered)	Sectoral approach, with emission factors collected from China Greenhouse Gas Inventory Research 2005 and 2008	CO_2_: industry, transport, agriculture, service, household, industrial processes, indirect emissions from electricity generation, forestry carbon sequestration. (Covered sectors for CH_4_, N_2_O, and fluorinated GHGs omitted here)	2015
^17^	GBA and surrounding Guangdong cities	CO_2_ emissions from energy consumption and industrial processes	Sectoral approach and reference approach, with emission factors collected from Liu *et al*.^31^	Industry, household, and industrial processes	2017
^18^	182 Chinese cities (including 12 Guangdong cities)	CO_2_ emissions from energy consumption and industrial processes	Sectoral approach, with emission factors collected from Liu *et al*.^31^	47 socioeconomic sectors belonged to primary industry, manufacturing industry, construction, service, and household	2010
^19^	GBA cities	CO_2_ emissions from impervious land (energy consumption) and cropland (fertilizers, machinery, agricultural film, and irrigation, CO_2_ absorption excluded)	Impervious land: emission factor method, with emission factors collected from Mahony^53^ and Yan *et al*.^54^, downscaled to 30m-grid with nighttime lighting; Cropland: emission factor method, with emission factors collected from Oak Ridge National Laboratory and Nanjing Agricultural University, downscaled to 30m-grid with NDVI data	Impervious land: residence and various production activities; Cropland: agricultural production	2001–2020
^20^	GBA and surrounding Guangdong cities	CO_2_ emissions from in-boundary energy consumption and industrial process	Sectoral approach, with emission factors collected from Liu *et al*.^31^	agriculture, forestry, fishery and livestock, industry, construction, service, transportation, and residence	2000–2019
^21^	Guangdong cities	CO_2_ emissions from energy consumption	Calculate-and-correct model, with emission factors collected from Wang *et al*.^55^	(Not specific)	2005–2017

Guangdong Province is the largest greenhouse gas (GHGs) emitter in southern China^22,23^, and CO_2_ was identified as the key contributor (92%) of total GHGs^24,25^. The CO_2_ emissions monitoring and urban climate change mitigation efforts were further elevated by the continuing urbanization and population growth in the region. This dataset provided comparable, transparent, and verifiable CO_2_ emissions inventories for nine GBA cities and twelve surrounding cities. The inventories covered 17 types of fossil fuel and 47 socioeconomic sectors, which were consistent with China’s national and provincial inventories.

The dataset supports the refinement of low-carbon strategies and the design of sustainable development policies at city-level. Consistent city-level emission estimates would facilitate multi-scale and inter-city carbon mitigation evaluation and comparative studies. Detailed sectoral and energy-specific emissions could be used for city-level studies focusing on mitigation pathways and related policy making.

## Methods

### Emission scope

This study followed the Intergovernmental Panel on Climate Change (IPCC) guidance^26^ to estimate in-boundary CO_2_ emissions from fossil fuel combustion and industry processes of prefectural-level cities in Guangdong Province, 2000–2022. Seventeen types of fossil fuel consumption (Table 2), 47 socioeconomic sectors (Table 3), and four types of industrial processes were considered from the production side. The emissions from electricity and heat production are calculated through primary energy inputs, without considering imports outside the administrative territorial boundary. Energy losses from transport and transformation processes, or used as chemical raw material, were removed from energy consumption to avoid double-counting.

**Table 2:** Emission factors of fossil fuels.

Fuel types		*NCV*_*j*_	*CC*_*i*_
		PJ/10^4^ t, 10^8^m^3^	tC/TJ
Coal	Raw coal	0.21	26.32
	Cleaned coal	0.26	26.32
	Other washed coal	0.15	26.32
	Briquette	0.18	26.32
	Coke	0.28	31.38
	Coke over gas	1.61	21.49
	Other gas	0.83	21.49
	Other coking products	0.28	27.45
Oil	Crude oil	0.43	20.08
	Gasoline	0.44	18.90
	Kerosene	0.44	19.60
	Diesel oil	0.43	20.20
	Fuel oil	0.43	21.10
	Other petroleum products	0.51	17.2
	LPG	0.47	20.00
	Refinery gas	0.43	20.20
Gas	Nature gas	3.89	15.32

**Table 3:** Socioeconomics sectors and category.

Socioeconomic sectors	Category	
Farming, Forestry, Animal Husbandry, Fishery and Water Conservancy	Farming sector	
Coal Mining and Dressing	Energy production	Manufacturing industries
Petroleum and Natural Gas Extraction		
Ferrous Metals Mining and Dressing		
Nonferrous Metals Mining and Dressing		
Nonmetal Minerals Mining and Dressing		
Other Minerals Mining and Dressing		
Petroleum Processing and Coking		
Production and Supply of Electric Power, Steam and Hot Water		
Production and Supply of Gas		
Logging and Transport of Wood and Bamboo	Light manufacturing	
Food Processing		
Food Production		
Beverage Production		
Tobacco Processing		
Textile Industry		
Garments and Other Fiber Products		
Leather, Furs, Down and Related Products		
Timber Processing, Bamboo, Cane, Palm Fiber & Straw Products		
Furniture Manufacturing		
Papermaking and Paper Products		
Printing and Record Medium Reproduction		
Cultural, Educational and Sports Articles		
Medical and Pharmaceutical Products		
Raw Chemical Materials and Chemical Products	Heavy manufacturing	
Chemical Fiber		
Rubber Products		
Plastic Products		
Nonmetal Mineral Products		
Smelting and Pressing of Ferrous Metals		
Smelting and Pressing of Nonferrous Metals		
Metal Products		
Ordinary Machinery		
Equipment for Special Purposes		
Transportation Equipment		
Production and Supply of Electric Power, Steam and Hot Water		
Electric Equipment and Machinery	High-tech industry	
Electronic and Telecommunications Equipment		
Instruments, Meters, Cultural and Office Machinery		
Other Manufacturing Industry		
Scrap and waste		
Construction	Construction	
Transportation, Storage, Post and Telecommunication Services	Services sectors	
Wholesale, Retail Trade and Catering Services		
Others		
Urban	Residential usage	
Rural		

### Emission calculation and inventory construction

**Fig. 2:**
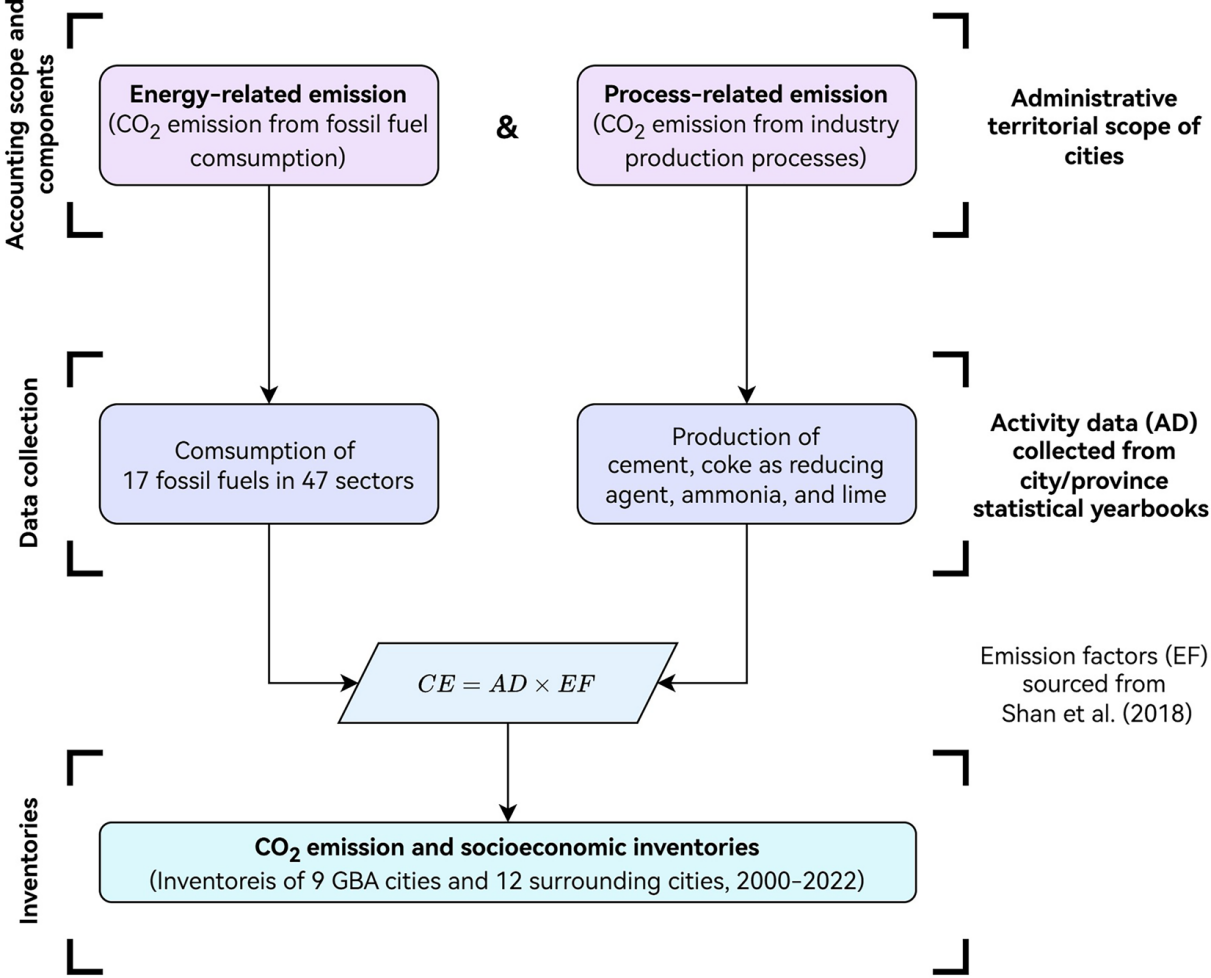
Diagram of CO_2_ emission inventories construction for GBA and surrounding cities.

This study constructed the CO_2_ emission inventories based on a uniform carbon emission accounting framework (Fig. 2) proposed by our previous work^27,28^. This study considered 17 types of energy, which can generally be categorized as coal, oil, and natural gas (Table 2). The inventories also incorporated emissions from four key industrial processes, including the production of cement, coke as a reducing agent, ammonia, and lime, which together contribute more than 95% of China’s process-related emissions^29^.

Energy-related CO_2_ emissions (*CE*_*e*_) were calculated based on the mass balance of fossil fuel consumption converted to CO_2_ emissions (Eq. 1).

1 $$C{E}_{e}=\sum {AD}\times {EF}=\mathop{\sum }\limits_{i=1}^{17}\mathop{\sum }\limits_{j=1}^{47}{{AD}}_{{ij}}\times {{NCV}}_{j}\times {{CC}}_{j}\times {O}_{{ij}}$$

where, *i* and *j* denoted the energy types and socioeconomic sector, respectively; *AD* referred to the activity data (i.e., fossil fuel consumption); ${NC}{V}_{j},C{C}_{j}$ and ${O}_{{ij}}$ represented three emission factors (*EF*), namely, net caloric value in the ${j}^{{th}}$ sector, carbon content in the ${j}^{{th}}$ sector, and oxygenation efficiency of ${i}^{{th}}$ energy type in ${j}^{{th}}$ sector. These emission factors were collected from our previous work^30^ and listed in Table 2.

Process-related emissions (${{CE}}_{p}$) were produced during chemical reactions in industrial processes. They were estimated using Eq. 2.

2 $${{CE}}_{p}=\mathop{\sum }\limits_{t=1}^{4}{{AD}}_{t}\times {{CE}}_{t}$$

where, ${{AD}}_{t}$ and ${{CE}}_{t}$ denoted the activity data (i.e., production of the industrial products) and the corresponding emission factor of industrial process of product *t*, respectively. The emission factor for cement and lime production were sourced from Liu *et al*.^31^ and Shan *et al*.^32^, respectively, and the rest of the emission factors were sourced from IPCC^26^.

Activity data of fossil fuels were collected from the Energy Balance Tables (EBTs), which provided the transformation and final consumption of each fuel^27,28,33^. The EBTs for Guangzhou (2000–2013), Qingyuan (2005–2014), and Yangjiang (2006–2022) were collected from the city’s statistical yearbooks^34–36^. For other cities and individual years without EBTs, Guangdong provincial EBTs sourced from national energy statistical yearbooks^37^ were scaled down to the city-level by the city’s share of the sector’s GDP and population. Energy consumption data were missing in Dongguan (2000–2013), Jiangmen (2004), Shenzhen (2004, 2006, and 2007), Zhongshan (2004), and Zhuhai (2000), and their energy consumption data were derived from the industry’s value-added from adjacent years. Due to data limitations, statistics from Hong Kong and Macao could not be included in this accounting framework. Emissions data for the two cities from 2000 to 2022 were sourced from the Emissions Database for Global Atmospheric Research (EDGAR) dataset version 8.0^38–40^, and appended as supplementary references to ensure the completeness of the inventory. The EDGAR dataset and our inventories adhered to the IPCC guidelines for emission estimations.

### Socioeconomic data

Data on population and GDP of 23 cities were collected from each city’s statistical yearbook. Detailed sources could be found in our dataset at Figshare^41^. The carbon emissions per unit of GDP and per capita in the inventory are derived using the population and GDP data.

## Data Records

The datasets consisted of CO_2_ emission inventories and socioeconomic data for the GBA and surrounding Guangdong cities, spanning from 2000 to 2022. The dataset has been made available at Figshare^41^. All inventories were organized in Microsoft Excel spreadsheets using a uniform structure. The carbon emission inventories were arranged as follows:

1. Summed CO_2_ emissions year-by-year at the city level [“Emission inventory.xlsx”, in sheet “Overview”];
2. Detailed CO_2_ emissions by 47 industry sectors [“Emission inventory.xlsx”, in sheet “CityEmission_byEnergy”] and by 17 energy types for each city [“Emission inventory.xlsx”, in sheet “CityEmission_bySector”]. Detailed emission data for Dongguan (2000–2013), Shenzhen (2004, 2006, and 2007), Jiangmen (2004), Zhuhai (2000), and Zhongshan (2004) were unavailable due to limited data accessibility.

Apart from the emission inventories, the socioeconomic data were compiled as a reference for the users. To make the records comparable across the year, the constant price of 2022 was applied to estimate GDP in chained volume. They were arranged in a single Excel file and recorded as follows:

1. Year-end population at city-level, in 10 thousand person [“Socioeconomic data.xlsx”, in sheet “Population”];
2. GDP in chained (2022) volume at the city-level, in 100 million Renminbi (RMB) [“Socioeconomic data”, in sheet “Gross Domestic Product”];
3. Price deflators of GDP (year 2022 = 100) at the city level [“Socioeconomic data.xlsx”, in sheet “Gross Domestic Product”].

## Technical Validation

### Statistical analysis

**Fig. 3:**
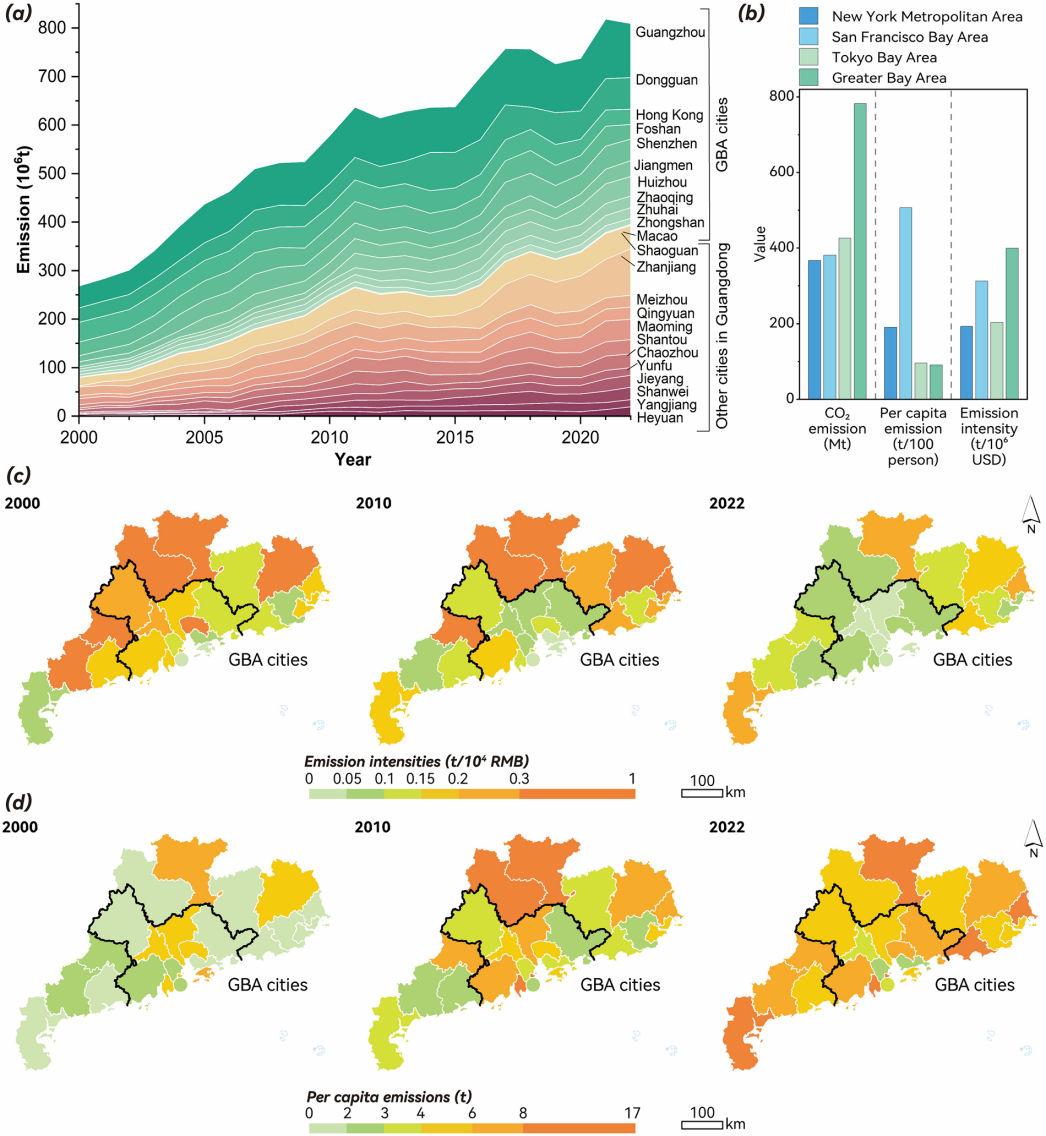
CO_2_ emissions of GBA and surrounding cities. (**a**) CO_2_ emission trend 2000–2022; (**b**) comparison of socioeconomic emissions across global bay areas; (**c**) CO_2_ emission intensity; (**d**) CO_2_ emissions per capita. Note that Macao is enlarged in size to make it visible on maps in (**c**) and (**d**).

Figure 3a illustrated the temporal evolution of the emissions in the GBA and surrounding cities from 2000 to 2022. Over the 23-year period, the CO_2_ emissions have increased at an average of 5.23% per year, reaching a maximum of 819 million tons in 2021. A rapid increase occurred during 2000–2007 with an annual growth rate of 9.65%, and surrounding cities grew 3.91% faster than GBA cities. Growth rates fluctuated after 2008 and slowed down to 2.97% after 2011. The slowdown in growth is attributed to fossil fuel reduction policies and technological innovations (e.g., clean energy promotion in energy production, industrial manufacture, transportation, and residence^42^), which lowered carbon intensity and emissions^20^. From 2020 to 2021, there was a 10.97% surge as energy demand rebounded following the COVID-19 pandemic, aligning with the national trend of economic recovery^43,44^.

During the period 2000–2022, the average emission intensity (ratio of total CO_2_ emissions to GDP) of GBA cities dropped from 0.24 to 0.10 t/10^4^ CNY (Fig. 3c). In terms of per capita CO_2_ emissions (Fig. 3d), eight cities (i.e., Qingyuan, Foshan, Guangzhou, Hong Kong, Shenzhen, Meizhou, Yunfu, and Zhongshan) showed decreasing trends from 2010 to 2022, but the average of GBA and surrounding cities increased from 4.91 to 5.13 t/capita. Despite the overall increasing trend, 12 cities that accounted for 62% region’s population had per capita CO_2_ emissions in 2022 lower than the European Union average (6.1 t/capita^45^). In comparison with other global bay areas (Fig. 3b), the GBA had the highest total emissions but the lowest per capita emissions. The 2022 emission intensity of GBA (396 t/10^6^USD) was comparable to that of the San Francisco Bay Area (331 t/10^6^USD^4,46^), as high-tech industries and service sectors dominated both bay areas^4,47^. These patterns were consistent with previous findings that GBA and the surrounding cities had made carbon decoupling progress through improving energy efficiency and industrial structure^48^, thus demonstrating the robustness of these inventories.

### Uncertainties

**Fig. 4:**
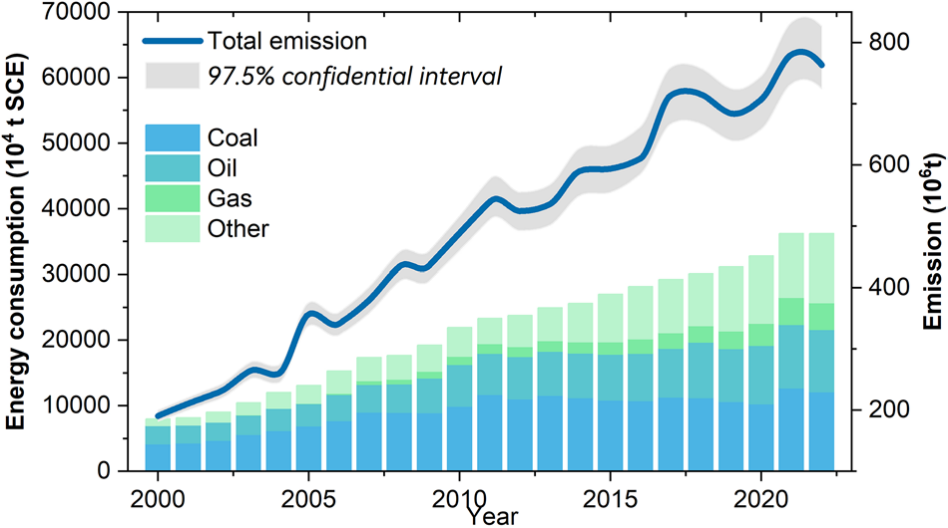
Energy consumption and total CO_2_ emissions in GBA and surrounding cities, 2000–2022.

The uncertainties of the inventories were mainly introduced from the activity data and emission factors^49,50^. Industrial process-related carbon emissions were not considered due to their relatively small share of total emissions (<9%) and usually have low uncertainty^28,31^. Uncertainties in energy-related carbon emissions were calculated using the Monte Carlo method recommended by the IPCC^26^. Due to data limitations, we assumed that both fossil fuel consumption and emission factors followed normal distributions, and coefficient of variation (CV, defined as the standard deviation divided by the mean) was set to 0.03 for coal, 0.01 for oil, and 0.02 for natural gas, and fossil fuel consumption have CV ranged from 5% to 30% depending on the sector^31^. Assuming both the fossil fuel consumption data and emission factors followed normal distributions^31^, their uncertainties were evaluated through 20,000 simulations, and a 97.5% confidence interval was estimated. The annual uncertainties of the CO_2_ emission estimations laid within the interval of [−13.07%, 13.07%] (Fig. 4). The largest uncertainty was observed from Shantou in 2018 ([−10.53%, 10.53%]), while the smallest uncertainty was from Chaozhou in 2000 ([−0.64%, 0.64%]).

### Comparison with existing work

Publicly available datasets on city-level carbon dioxide emissions for the GBA and surrounding Guangdong cities are currently rare. We collected comparable emission estimates for this area from existing literature to facilitate data comparison (Table 4). Shan *et al*.^18^, Luo *et al*.^20^, and Lin *et al*.^17^ employed the sectoral approach to estimate carbon dioxide emissions in Guangdong at the city level. The total emissions from Luo *et al*. are close to our estimations, with a gap ranging between 19.2% (2017) and 0.6% (2009). CO_2_ emissions for Guangzhou, Shenzhen, Zhuhai, and Shantou from Shan *et al*. were very close to our estimations, with a range from 1.86% to 4.12%. Variance existed in cities reliant on energy and the manufacturing sectors (e.g., Shaoguan, Maoming, Yangjiang, and Huizhou). The quality of activity data for these cities causes these variances. This study has updated the activity data based on the latest available statistical releases. Complete energy balance tables and detailed statistical data beyond major cities are essential for accurate emissions estimation. Lin *et al*. provided 2017 emissions for 21 Guangdong cities, categorized by energy consumption, industrial processes, and household energy use. While differences in sectoral categorization hindered direct comparisons at the sectoral level, total emissions of most cities are consistent with our 2017 inventory results, except for Guangzhou, Dongguan, Shenzhen, Yangjiang, and Maoming (>25% difference). Our estimations updated emission factors to cover 17 energy sources, while Lin *et al*. only considered coal, oil, and natural gas. This disparity may contribute to the differences.

**Table 4:** Comparisons of emission accounting results with existing works.

City-by-city comparison with Shan *et al*. and Lin *et al*. (Totals of cities)							Year-by-year comparison with Luo *et al*. (Totals of GBA and surrounding Guangdong cities)			
City	This dataset (2010)	Shan *et al*.(2010)	RPD *	This dataset (2017)	Lin *et al*. (2017)	RPD *	Year	This dataset	Luo *et al*. **	RPD *
Guangzhou	98.65	100.50	1.86%	116.2	65.3	56.1%	2000	268.5	252.6	6.1%
Dongguan	47.00	—	—	72.0	43.5	49.4%	2001	283.7	265.4	6.7%
Foshan	37.55	—	—	43.9	48.1	9.2%	2002	301.2	287.1	4.8%
Shenzhen	37.09	38.65	4.12%	49.7	31.3	45.6%	2003	340.9	321.5	5.9%
Jiangmen	26.79	30.94	14.39%	25.4	26.4	3.9%	2004	391.1	361.1	8.0%
Huizhou	11.76	23.59	66.91%	26.1	29.9	13.5%	2005	436.8	407.0	7.1%
Zhaoqing	14.33	—	—	28.3	22.3	23.8%	2006	463.3	444.0	4.3%
Zhuhai	12.76	13.00	1.91%	19.0	17.3	9.5%	2007	509.8	484.8	5.0%
Zhongshan	10.12	17.21	51.87%	14.0	11.3	21.8%	2008	522.3	492.5	5.9%
Shaoguan	39.96	22.73	54.99%	46.7	59.7	24.3%	2009	524.8	521.9	0.6%
Zhanjiang	24.48	—	—	60.4	48.3	22.4%	2010	578.3	566.5	2.1%
Meizhou	29.41	—	—	22.7	21.9	3.6%	2011	636.9	604.8	5.2%
Qingyuan	31.81	—	—	29.3	25.9	12.2%	2012	615.0	585.6	4.9%
Maoming	14.75	27.80	61.34%	28.2	18.5	41.5%	2013	628.3	570.3	9.7%
Shantou	24.85	24.43	1.70%	28.5	23.7	18.3%	2014	636.7	574.2	10.3%
Chaozhou	17.26	—	—	24.0	20.1	17.7%	2015	638.0	586.9	8.3%
Yunfu	16.02	17.86	10.90%	14.1	13.0	7.6%	2016	699.2	602.2	14.9%
Jieyang	13.58	—	—	17.4	17.3	1.0%	2017	757.6	625.2	19.1%
Shanwei	10.18	—	—	23.4	21.9	6.8%	2018	756.8	632.9	17.8%
Yangjiang	7.13	15.83	75.85%	12.4	25.1	67.9%	2019	726.3	625.2	15.0%
Heyuan	10.15	9.41	7.55%	11.4	13.2	14.7%				

*RPD (Relative Percentage Difference) was calculated using the formula: ${RPD}=\left|{x}_{1}-{x}_{2}\right|/(\frac{{x}_{1}+{x}_{2}}{2})$, where ${x}_{1}$ and ${x}_{2}$ denote the values being compared.

**The data were digitized from Figure 5a of Luo *et al*. due to the unavailability of raw/tabulated data from the original study.

### Limitations and future work

Our inventories have some limitations that may lead to uncertainty. (1) Hong Kong and Macao could not be directly incorporated into the accounting framework. Future work will leverage bottom-up statistical data and calibrated general observations (e.g., satellite imagery) to provide more accurate CO_2_ emission estimates for these cities. (2) Renewable energies (e.g., solar power, wind power, and hydropower) were assumed as zero-carbon energy sources in this study, and the emissions from manufacturing are excluded. Indirect emissions along the supply chain will be incorporated. (3) This dataset only covers CO_2_ emissions. Agricultural production is the prominent contributor to non-CO_2_ greenhouse gases (e.g., CH_4_ and N_2_O). More efforts are needed to incorporate non-CO_2_ greenhouse gases into the accounting framework by leveraging process-based models and the satellite-based inversion method.

## Data Availability

The dataset is available at Figshare^41^ (10.6084/m9.figshare.28235681).

## Code availability

The MATLAB codes used to generate the emissions inventories are published in the Supplementary Information section of our previous work^18^.
